# Quality of life, symptoms, and sleep quality of elderly with end-stage renal disease receiving conservative management: a systematic review

**DOI:** 10.1186/s12955-019-1146-5

**Published:** 2019-05-03

**Authors:** Qingli Ren, Qifang Shi, Tong Ma, Jing Wang, Qian Li, Xiaomei Li

**Affiliations:** School of Nursing, Xi’an Jiaotong University, Xi’an, China

**Keywords:** Quality of life, Symptoms, Sleep quality, Conservative management, End-stage renal disease, Systematic review

## Abstract

**Background:**

Older patients with end-stage renal disease (ESRD) have experienced diminished quality of life and debilitating symptoms. Conservative management may be a potential treatment option. Currently, limited studies have been conducted about the main outcome of conservative management, including quality of life, symptoms and sleep quality. The aim of this systematic review was to examine the quality of life, symptoms and sleep quality of elderly patients with ESRD undergoing conservative management.

**Methods:**

Evidence-based medicine database (JBI and Cochrane) and original literature database (PubMed, Medline, EMbase, Web of Science) were searched up to March 12, 2018. The quality of included papers was evaluated with the Newcastle-Ottawa Scale.

**Results:**

Eight studies met the inclusion criteria. The total of 1229 patients were involved with an average age of 60.6 ~ 82 years. Patients choosing conservative management were older and more functionally impaired compared to those opting for dialysis. 55% patients undergoing conservative management had stable or improved quality of life and symptoms in prospective cohort study. However, the results revealed that there were no significant differences in quality of life and symptom between conservative management and renal replacement therapy. Only one study assessed quality of life of older patients using SF-36, with a lower score in physical health subscale of conservative management patients than those of renal replacement therapy. Although more than 40% of the patients had poor sleep quality, no significant difference was found between conservative management and renal replacement therapy. Sleep disorders were associated with fatigue and other symptoms.

**Conclusions:**

Although there is a limited literature, conservative management is likely to improve quality of life and alleviate symptoms of end-stage renal disease patients with considerable clinical implications mainly in elderly patients. Future study should pay more attention to the various treatment outcomes of conservative management, providing abundant evidence.

**Electronic supplementary material:**

The online version of this article (10.1186/s12955-019-1146-5) contains supplementary material, which is available to authorized users.

## Background

On a global scale, chronic kidney disease (CKD) is becoming more and more common, with a prevalence of 11% in developed countries [1]. And the prevalence of the elderly is 3 to 13 times higher than that of young people [2–4]. More than a quarter of CKD patients who reach to end-stage renal disease (ESRD) are older patients (aged ≥75) [5]. Patients with CKD experience significant lifestyle changes which seriously affect the physical or mental health. Especially for elderly patients, they have multiple comorbidities and complications. Their quality of life (QOL) is rapidly declining, and symptom burden is rapidly escalating [6–8].The current treatment for patients with ESRD is renal replacement therapy (RRT), mainly dialysis. However, dialysis has not always been considered suitable. A study conducted in 2016 found that RRT may not always benefit ESRD patients [9]. On dialysis initiation, the elderly have increased morbidity and mortality, along with a variety of post-dialysis symptoms and comorbidities, such as disequilibrium syndrome [10].

An increasing amount of evidence indicated that the effect of conservative management (CM) is similar to or higher than that of dialysis in terms of survival or QOL [11, 12]. A few studies also stated that CM can ensure patient’s activities of daily living with minimal restrictions while reducing the economic burden [13, 14]. Nevertheless, it is still very difficult for patients with end-stage renal disease to choose dialysis or conservative management.

Conservative management can be identified as a potential treatment option, but lack of clear definition. At the KDIGO Controversies Conference, experts have suggested conservative management defined as “*an approach that improves the quality of life of patients and their families facing the problems associated with life-threatening illness, through the prevention and relief of suffering by means of early identification and impeccable assessment and treatment of pain and other problems, physical, psychosocial and spiritual* [15].” A series of recent systematic reviews targeted survival analysis or how to make a decision for elderly patients undergoing RRT or CM [6, 16, 17]. Another recent review of 12 studies (*n* = 11,515 patients) focused on which therapies (dialysis vs. conservative management) would improve life expectancy [18]. Despite the evidence that CM may be beneficial for elderly patients with ESRD, concerns have remained. A critical question for researches is the lack of analyzable data regarding quality of life, symptoms [19]. QOL has been considered as a powerful predictor of treatment outcomes [5, 20]. Only a few studies have addressed QOL in ESRD patients receiving conservative management, especially lack of the accessing for QOL in CM of older patients vs. other modalities [7, 21–25]. In addition, symptoms and sleep quality are important treatment outcomes and can also seriously affect the patients’ QOL, but few studies have shown their impact on elderly undergoing CM. Therefore, this systematic review was conducted to evaluate QOL, symptoms and sleep quality of elderly patients with ESRD undergoing conservative management.

## Method

### Eligibility criteria

The literature involving quality of life among ESRD patients undergoing CM is limited. A decision was made to hold all the literature containing: 1) meta-analyses or systematical reviews for reference list hand-searches; and 2) clinical trials for screening. Hence, we analyzed completed reviews for gaining sufficient information and additional papers.

Inclusion criteria: 1) elderly patients aged ≥60 years with ESRD or CKD stage 5; 2) one group patients receiving CM/palliative/hospice care; 3) at least one control group (no specific limit for the control group); 4) QOL/HRQOL, symptoms or symptom burden, sleep quality as one of the treatment outcomes; 5) cohort study, case-control study or randomized controlled study (RCT); 6) primary research; 7) English language. Meta analyses and systematic reviews, duplicative papers, opinion papers, unobtainable and unusable data were excluded.

### Information sources and search

This search included Evidence-based medicine database (JBI and Cochrane) and original literature database (PubMed, Medline, Web of Science, EMbase, Wanfang and CNKI). Databases were searched up to March 12, 2018. Mesh terms, EMTREE terms, key words and item words were used to search included chronic kidney disease (CKD) or chronic kidney failure or end-stage renal disease (ESRD), palliative care or conservative management or conservative care, health related quality of life or health care quality or quality of life (QOL), sleep*, symptom* or symptom burden. Additional file 1: Appendix A shows the concrete search strategies.

The combined searches yielded 1336 papers as of March 12, 2018. All papers were imported into EndNote and 834 duplicates were removed mechanically. The remaining papers (502) were imported into an Endnote library for preliminary screening according to the inclusion/exclusion criteria performed by the team.

### Study selection

**Fig. 1:**
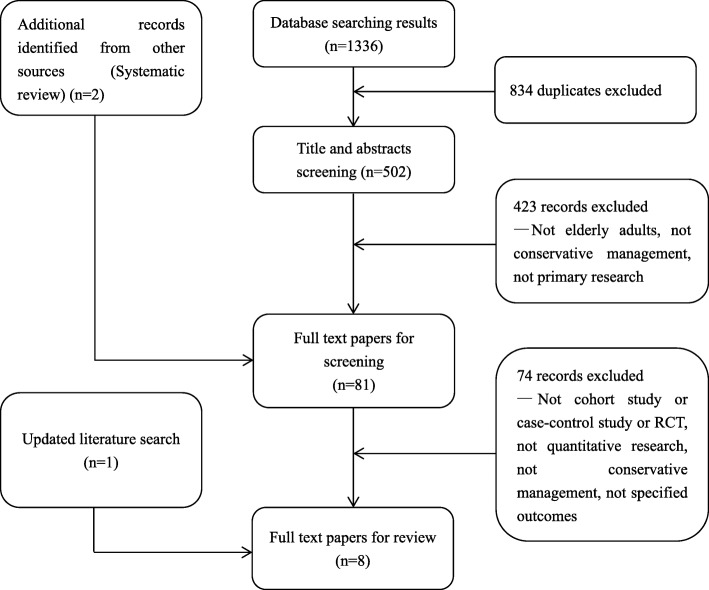
Flowchart of study identification

For selecting the final papers, a two-step screening process was conducted. In the first screening, all titles and abstracts were reviewed by two independent reviewers (Q.R. & T.M.). For retaining as many papers as possible, we decided to retain any ambiguous papers for the next step. In the second screening (full-text screening), we reviewed the full-text of the 81 papers and some supplements were made according to the references. A third reviewer (Shi) was consulted if there was no consensus. (See Fig. 1).

### Quality assessment

The Newcastle-Ottawa Scale (NOS) with some modifications to match the needs of this study was used to evaluate the quality of included papers (Table 1). It judged on three broad perspectives and allocates a maximum of 9 points, including quality of selection (score 0–4), comparability (score 0–2), and outcome (score 0–3) [26]. Quality of selection means the selection of the study group, which has four questions. Comparability means the comparability of study groups, which has two questions. Outcome means the ascertainment of outcome of interest, which has three questions. The details were shown in Table 1. Scores of 3, 6, 9 points were interpreted as poor, fair, or good quality. It has been established validity and inter-rater reliability.

**Table 1:** Check List for Quality Assessment and Scoring of Nonrandomized Studies

Check list	
Selection	
1. Is the case definition adequate? (one star for independent validation)	
2. Representativeness of the cases (one star for obviously representative series of cases)	
3. List inclusion and exclusion criteria for exposed and unexposed subjects. (one star for clear description)	
4. Describe any assessments undertaken for quality assurance purposes. (one star for comparability or test-retest of primary outcome measurements)	
Comparability	
5. Explain any patient exclusions from analysis. (one star for yes)	
6. Describe how confounding was assessed and/or controlled. (one star for yes)	
Outcome assessment	
7. Determination of results. (one star for secure records)	
8. Summarize patient response rates and completeness of data collection. (one star for yes)	
9. Adequacy of follow up. (one star if follow-up≥80% or effective response rate ≥ 80%)	

Two authors (Q.R. & T.M.) independently evaluated the quality of each study. Any inconsistent in instances were resolved by consensus with a third reviewer. Then the inter-rater reliability of reviewers was assessed with the weighted kappa coefficient. According to the Landis and Koch’s guidelines, kappa index were interpreted as slight agreement (0.0–0.2), fair agreement (0.21–0.40), moderate agreement (0.41–0.6), substantial agreement (0.61–0.80), and almost perfect agreement (0.81–1) [27].

### Data extraction

We extracted following data from eligible papers: author, country of origin, years of publication, study design, sample size, type of patients, age of patients, gender of patients, major exclusion criteria. The following data also were extracted: the scale, related data and descriptions for using to measure HRQOL or QOL, symptoms and symptoms burden, sleep quality. Two reviewers independently conducted this process. In case of controversy, the decision was taken through consultation.

## Results

### The search results

Eight studies met the inclusion criteria [7, 21–25, 28, 29]. Six addressed QOL of elderly patients undergoing CM [7, 21–23, 25, 29], Five addressed symptom of elderly patients undergoing CM [22, 24, 25, 28, 29], and four evaluated the sleep of elderly patients undergoing CM [22, 24, 28, 29]. All were cohort studies [7, 21–25, 28, 29] with six using prospective data [7, 21–25, 29] and two using retrospective data [23, 28]. Only two studies evaluated all of these factors and met the criteria [22, 29]. No publication was a randomized controlled study. All eligible studies had one or more control groups [7, 21–25, 28, 29] (five RRT, one predialysis and two terminal malignancy, respectively). In addition, the total of 1229 patients were included in this systematic review with an average age of 60.6 ~ 82 years. (See Table 2 & Table 3).

**Table 2:** Studies of older Patients Undergoing Conservative Management (CM): Quality of Life (QOL)

Author	Country	Study type	Type of interventio-n	Participa-nts, n	Age, mean(SD)	Dropout Rate, %	Exclusion Criteria	Setting	Outcome Measures	Results	Quality assess-ment
Brown MA et al.(2015) [25]	Australia	Prospective cohort study	Nondialysis, predialysis	467	74.5	15.4	NA	Renal clinics	SF-36	Of the nondialysis patients, 58% had stable or improved QOL.	8
Seow et al.(2013) [7]	Singapore	Prospective cohort study	CM, RRT	110	74.5	8.2	NA	Renal wards,outpatient clinics	KDQOL-SF	No difference for QOL between CM and RRT	8
Da Silva-Gane et al.(2012) [21]	United Kingdom	Prospective cohort study	CM, hemodialysis	170	77.5 (6.5) 60.6 (14.9)	0	Lacking capacity or with poor under- standing of English	Low-clearance clinics	SF-36	Mental health of CKM patients was significantly lower than that of HD and PD patients.	8
Yong et al.(2009) [22]	Hong Kong	Prospective cohort study	Palliative-care, Dialysis	191	61.9 (12.3)	6.3	Cognitive impairment or known psychiatric illness	Hospital	SF-36	The dialysis group scored significantly lower than Hong Kong population.	8
De Biase et al. (2008) [23]	Italy	Retrospective cohort study	CT, hemodialysis	16	80.45	0	NA	Outpatients clinic	SF-36	Similar QOL for two groups, though the patients on CT had numerous comorbidities and their functional levels were more severe.	7
Saini et al. (2006) [29]	United Kingdom	Prospective cohort study	CM, terminal malignancy	22	65	0	Aged < 18 years, unclear themselves diagnosis and its implications.	Renal clinic	Euroqol EQ-5Q	Similar QOL for two groups.	7

Abbreviations: *CKM* conservative kidney management, *CT* Conservative treatment, *HD* hemodialysis, *PD* peritoneal dialysis, *CM* Conservative Management, *QOL* Quality of Life, *RRT* renal replacement therapy

**Table 3:** Studies of older Patients Undergoing Conservative Management (CM): Symptom

Author	Country	Study type	Type of intervention	Participants, n	Age(mean ± SD)	Dropout Rate,%	Exclusion Criteria	Setting	Outcome Measures	Results	Quality assessment
Wan Zukiman et al. (2017) [24]	Malaysia	Prospective cohort study	Nondialysed, RRT	187 (100 nondialysed, 87 RRT)	60.97 (13.89)	0	Pregnancy; presence of any type of acute psychiatric disorder; lack of capacity to give informed consent; inability to communicate fluently in Malay or English language; or illiteracy.	Nephrology department	DSI	No difference in the prevalence of symptom burden and severity between two groups.	7
Brown MA et al.(2015) [25]	Australia	Prospective cohort study	Nondialysis, predialysis	467	74.5	15.4	NA	Renal clinics	MSAS-SF	57% had stable or improved symptoms over 12 months for nondialysis patients.	8
Yong et al.(2009) [22]	Hong Kong	Prospective cohort study	Palliative-care, dialysis	191	61.9 (12.3)	6.3	Cognitive impairment or known psychiatric illness	Hospital	23 symptoms related to ESRD was assessed using NRS	No significant difference between the palliative care group and the dialysis group (*P* = 0.243)	8
Murtagh et al. (2007) [28]	United Kingdom	Retrospective cohort study	CM, Advanced cancer	66	82 (6.6)	0	Lacked capacity to consent to research participation	Renal units	MSAS-SF	Patients with ESRD have considerable symptom control needs, similar to advanced cancer populations.	6
Saini et al.(2006) [29]	United Kingdom	Prospective cohort study	CM, terminal malignancy	22	65	0	Aged< 18 years,unclear themselves diagnosis and its implications	Renal clinic	MSAS-SF	Similar symptom burden for two groups.	7

Abbreviations: *DSI* The 30-item Dialysis Symptom Index, *NRS* numerical rating scale, *MSAS-SF* Memorial Symptom Assessment Scale–Short Form, *POSs* the Patient Outcome Scale (symptom module)

Quality assessment of this study used NOS. The two reviewers were in complete agreement, and the agreement on quality score of the individual studies was substantial agreement (Weighted Kappa = 0.61). The results found that all eight studies scored 6 to 8 (average score: 7.4), indicating good quality for all studies. All eligible studies earned one star for being representativeness of the average age in this population, one star for ascertainment of exposure with structured interview. For assessment of outcome, all had secure record, and follow up rate > 80% with a relative complete follow-up.

### Older patients with ESRD undergoing CM: QOL

Of the six studies [7, 21–23, 25, 29] (Table 2) evaluating QOL of older patients on CM, four had a combination of elderly undergoing CM and RRT, the other two studies were based on predialysis patients and terminal malignancy patients respectively. The sample size of these studies was ranging from 16 to 467 patients. One study has showed that QOL of two groups indeed has significantly impaired compared to the sex- and age- adjusted Hong Kong population (*P* < 0.01) (Dialysis group vs. HK population, CM group vs. HK population, respectively) [22]. However, there is no direct comparison between CM group and dialysis group. The other two studies found that the total QOL scores of older patients with ESRD undergoing CM were similar to those of aged-matched undergoing RRT [7, 23]. A recent study also assessed QOL of older patients using SF-36, with a lower score in physical health subscale of CM patients than those of RRT and similar score in mental health subscale [21]. Besides, another study reported that older patients undergoing CM had stable or improved QOL [25]. For the physical health subscale, 21% patients had improved QOL and 16% had a stable QOL undergoing CM. For the mental health subscale, 53% patients undergoing CM had improved QOL and 5% had a stable QOL [25]. Especially to be mentioned, scores in every domain of SF-36 correlated inversely with the number of symptoms.

### Older patients with ESRD undergoing CM: symptoms

Of the five studies [22, 24, 25, 28, 29] (Table 3) examining symptoms of older patients on CM, four studies had a control group (two RRT, one predialysis and one terminal malignancy, respectively). Two of the studies were conducted in the United Kingdom [28, 29], one each in Malaysia [24], Australia [25] and Hong Kong [22]. Sample size was variable, ranging from 22 to 467 patients. The objective of all these studies was to investigate symptom prevalence and severity. The reported symptom burden of older patients in ESRD undergoing CM was similar to the advanced cancer patients [28, 29]. For elderly patients of ESRD, the five most common reported symptoms were fatigue, cold aversion, pruritus, lower torso weakness and dry skin. Some symptoms were more prevalent in the CM group such as worrying, decreased appetite, numbness, and leg swelling, whereas, skin changes, halitosis and sexual problem were more prevalent in the dialysis group [22, 24]. Overall, the patients reported comparable symptom burden between CM and dialysis [22, 24]. At the same time, another study also showed that 53% CM patients had improved symptoms compared with predialysis patients over 12-month follow-up [25].

### Older patients with ESRD undergoing CM: sleep quality

Four studies [22, 24, 28, 29] mentioned sleep-related as part of the symptoms in ESRD older patients undergoing CM. No research specifically focused on sleep quality has been retrieved. But these four studies found that more than 40% ESRD patients commonly experienced poor sleep quality, which were manifested as trouble staying asleep, trouble falling asleep [22, 24, 28, 29]. Further comparisons indicated that there was no significant difference between CM and dialysis in difficulty sleeping [22, 24]. And sleep disorders was associated with fatigue and other symptoms [22]. However, only four studies limit the generalization of results.

## Discussion

Although there are many studies addressing QOL and symptoms in ESRD patient, few have focused on them in elderly patients specifically, and even fewer have examined them in older patients with ESRD undergoing CM. Moreover, there is no research on the relevant aspects of Chinese mainland, and it is urgent to summarize the existing international studies to guide future research.

Although data was limited, we still found some interesting results. CM and RRT had similar effects for improving QOL of older ESRD patients, with considerable clinical implications in these individuals. One study found that 58% patients had stable or improved QOL undergoing CM [25]. The reason may be that the patients on CM were managed by a multidisciplinary team. In this team, a palliative care specialist or a senior nurse can help improve patient’s QOL through the prevention and relief of suffering by means of early identification and impeccable assessment and treatment of pain and other problems, physical, psychosocial and spiritual. A dietician or social worker can also give support as needed. This can be confirmed by the following study: at first visit, it showed that the score of physical health had a significant difference in the two groups, with a lower score in the CM group than in the predialysis group. After a 12-month follow up, the significant difference of physical health score was eliminated [25]. A possible explanation for this change was that patients received more disease management information in the CM from the multidisciplinary team and can improve their quality of life over time. Thus, the overall QOL scores and mental health status of older patients undergoing CM were similar to the age-matched elderly patients undergoing RRT [7, 23]. Moreover, the result of another study showed that the trajectory of Mental Component Summary scores had more fluctuations both before and after RRT in the RRT group than the CM group [7]. This may be due to the difficulty adaption of patients after dialysis initiation [30, 31]. Therefore, it is probably that CM may be a treatment option, especially for older, high comorbid and complication patients. Even so, comparison between CM and RRT requires further research in strict design. The palliative care specialist or senior nurse maybe help the patients make clinical decision combined with the physical health of patients, goals of patients and families and so on. More researches are needed about what kind of patient is suitable for CM. It also can focus on how to identify the expectations of patients and their caregivers, and how to use the team management and individualized services in combination with existing resources to achieve the optimal outcome for patients.

Simultaneously, this study reviewed all articles about symptoms for patients with ESRD undergoing CM. As well known, the ESRD patients had high symptom burden [22, 24], which was similar with advanced cancer patients [28, 29]. Although the patients in both groups reported comparable symptom [22, 24], there were still differences between CM and RRT. For example, decreased appetite and leg swelling were more prevalent in CM group. This may be due to that water cannot be completely excreted from the body through drugs or other ways. Skin changes, halitosis and sexual problem were more prevalent in dialysis group. This may be related to acid-base balance disturbance caused by dialysis. Therefore, dialysis and conservative management have their own advantages or disadvantages in symptom management. Especially for the elderly, they have multiple comorbidities and complications. Symptom management is very difficult. It is worthy of recognition that 53% patients in CM group have stable or improved symptoms [25]. In future, clinical nurses could teach elderly ESRD patients more knowledge and skills in symptom management combine their mental state, such as how to management the intake of water. Due to the limitations of observational studies and unreported treatment time, it is unlikely to draw generalizable conclusions which treatment is better. More researches are needed. In addition, two studies focused on a period of time before the patient’s death [24, 28]. A systematic review suggested the need to delay initiation of dialysis [12]. It should be considered whether further staging research is necessary.

Literature was very limited concerning sleeping in older patients in ESRD undergoing CM, with uniformly high difficulty sleeping [22, 24, 28, 29]. Sleep disorders were associated with fatigue and other symptoms [22]. Some researches mentioned that therapies may impact sleep quality [32, 33]. Dialysis initiation may have negative impact on sleep quality. However, no significant difference was found between CM and dialysis in difficulty sleeping [22, 24]. Proper activity or return to society could be beneficial for patients’ sleep quality [33]. In addition, qualitative research revealed that older patients were willing to sacrifice potential survival advantages for better autonomy and quality of life [34], but lack relevant evidence. Therefore, rigorous researches into outcomes such as quality of life, symptoms and sleep quality are needed, which are important for older patients.

Some research mentioned comorbidity was an important factor in determining the benefits of different treatment modalities [17], but lack of specialized data. Whether the existing differences have clinical value needs further consideration.

The major strength of our study was to review the current literature on the QOL, symptoms and sleep quality of patients with conservative management in English language. And QOL is a powerful predictor of treatment outcomes. Symptoms and sleep quality play important roles in selecting treatment [22], and also have closely associated with QOL [22, 24, 28, 29]. This study included a broad range of patients and didn’t restrict the type of control group. We furthest have summed up the QOL of patients with conservative management. Owing to the heterogeneity of study design, the main limitation of this study was lacking of quantitative data synthesis and high-quality clinical studies such as RCT. There may be bias in reporting because the number of patients under different treatment is different.

In addition, there is no literature on conservative management in mainland China. Regarding to individuals, family burden and social-economic factors, this group needs to be noticed. On the basis of this study, our team will investigate the situation of conservative management population in China and conduct large cohort studies with rigorous design.

## Conclusion

Although there is limited literature, conservative management may have improved quality of life and alleviated symptoms of ESRD patients. It is reasonable to suggest that CM have considerable clinical implications mainly in elderly adults. However, every patient deserves to be well-informed of the potential benefits and possible adverse events of all options. Moving forward, it is needed to examine the QOL, symptoms and sleep quality of older patients with ESRD undergoing CM, including comparative studies with dialysis patients.

## Additional file

Additional file 1: **Appendix A.** Detailed search strategies. (DOCX 14 kb)

## Abbreviations

CKD: Chronic Kidney Disease
CKM: Conservative Kidney Management
CM: Conservative management
CT: Conservative Treatment
DSI: The 30-item Dialysis Symptom Index
ESRD: End-stage Renal Disease
HD: Hemodialysis
HRQOL: Health-related Quality of Life
MSAS-SF: Memorial Symptom Assessment Scale–Short Form
NA: None available
NOS: The Newcastle-Ottawa Scale
NRS: Numerical Rating Scale
PD: Peritoneal Dialysis
POSs: The Patient Outcome Scale (symptom module)
QOL: Quality of Life
RCT: Randomized Controlled Study
RRT: Renal Replacement Therapy
