# Supplementary material for: Quality of life, symptoms, and sleep quality of elderly with end-stage renal disease receiving conservative management: a systematic review
Source: Health Qual Life Outcomes. 2019 May 3;17:78. doi: 10.1186/s12955-019-1146-5 (PMC6500052; doi:10.1186/s12955-019-1146-5)
Supplement: Supplementary file 1 — Appendix A. Detailed search strategies. (DOCX 14 kb) [file 12955_2019_1146_MOESM1_ESM.docx]

**Appendix A. Detailed search strategies**

This search included Evidence-based medicine database (JBI and Cochrane) and original literature database (PubMed, Medline, EMbase, Web of Science). Databases were searched up to March 12, 2018.

Mesh terms, EMTREE terms, key words and item words were used to search included chronic kidney disease (CKD) or end-stage kidney disease (ESKD) or chronic kidney failure, palliative care, conservative management, conservative care, quality of life (QOL) or health care quality, sleep*, symptom* or symptom burden. All languages are limited to English searchable.

**EMbase**

(‘Chronic kidney disease’ OR ‘the End-stage Renal Disease’) and (‘conservative treatment’ OR ‘conservative care’ OR ‘Palliative care’ OR ‘end-of-life care’ OR ‘hospice care’ OR ‘hospice’ OR ‘Palliative Care’) and (‘quality of life’ OR ‘health care quality’) AND (‘symptom’ OR ‘symptoms’ OR ‘symptom burden’) AND (‘sleep’ OR ‘sleeping’ OR ‘sleep disorder’ OR ‘sleep dysfunction’)

**PubMed**

(((((symptom[All Fields] OR symptoms[All Fields] OR (symptom[All Fields] AND burden[All Fields])) OR (sleep*[All Fields]))AND ((((("quality of life"[MeSH Terms] OR "quality of life"[All Fields]) OR ("quality of health care"[MeSH Terms]) OR "quality of health care"[All Fields] OR "health care quality"[All Fields])) OR QOL [All Fields]) AND ((((((("conservative treatment"[MeSH Terms] OR "conservative treatment"[All Fields])) OR ("palliative care"[MeSH Terms] OR ("palliative"[All Fields]) OR "palliative care"[All Fields])) OR ("terminal care"[MeSH Terms] OR "terminal care"[All Fields] OR "end of life care"[All Fields] OR "hospice care"[MeSH Terms] OR ("hospice"[All Fields]) OR ("palliative care"[MeSH Terms] OR ("palliative"[All Fields]) OR "palliative care"[All Fields]))) AND (((OR "chronic kidney disease"[All Fields]) OR CKD[All Fields]) OR ("kidney failure, chronic"[MeSH Terms] OR "chronic kidney failure"[All Fields] OR ("end stage renal disease"[All Fields])) OR ESKD[All Fields])

**Web of Science**

(‘Chronic kidney disease’ OR ‘CKD’ OR ‘the End-stage Renal Disease’ OR ‘ESKD’) AND (‘conservative treatment’ OR ‘conservative care’ OR ‘Palliative care’ OR ‘end-of-life care’ OR ‘hospice care’ OR ‘hospice’ OR ‘Palliative Care’) AND (‘quality of life’ OR ‘health care quality’ OR ‘QOL’) AND (‘symptom’ OR ‘symptoms’ OR ‘symptom burden’) AND (‘sleep*’)

**Cochrane Library/JBI Library**

(Chronic kidney disease OR CKD OR the End-stage Renal Disease OR ESKD) AND (conservative treatment OR conservative care OR Palliative care OR end-of-life care OR hospice care OR hospice OR Palliative Care)

The combined searches yielded 1336 papers as of March 12, 2018. All papers were imported into EndNote and 834 duplicates were removed mechanically. The remaining papers (502) were imported into an Endnote library for preliminary screening according to the inclusion/exclusion criteria performed by the team.
